# Incidence of Severe and Nonsevere Pertussis Among HIV-Exposed and -Unexposed Zambian Infants Through 14 Weeks of Age: Results From the Southern Africa Mother Infant Pertussis Study (SAMIPS), a Longitudinal Birth Cohort Study

**DOI:** 10.1093/cid/ciw526

**Published:** 2016-11-02

**Authors:** Christopher J. Gill, Lawrence Mwananyanda, William MacLeod, Geoffrey Kwenda, Magdalene Mwale, Anna L. Williams, Kazungu Siazeele, Zhaoyan Yang, James Mwansa, Donald M. Thea

**Affiliations:** 1 Center for Global Health and Development; 2 Department of Global Health, Boston University School of Public Health, Massachusetts; 3 Zambia Center for Applied Health Research and Development; 4 Department of Biomedical Sciences, School of Medicine, University of Zambia, Lusaka; 5 Data Coordinating Center, Boston University School of Public Health, Massachusetts; 6 Department of Pathology and Microbiology, University Teaching Hospital, Lusaka, Zambia

**Keywords:** whooping cough, *Bordetella pertussis*, incidence, cohort study, prospective surveillance

## Abstract

***Background.*** Maternal vaccination with tetanus, reduced-dose diphtheria, and acellular pertussis vaccine (Tdap) could be an effective way of mitigating the high residual burden of infant morbidity and mortality caused by *Bordetella pertussis*. To better inform such interventions, we conducted a burden-of-disease study to determine the incidence of severe and nonsevere pertussis among a population of Zambian infants.

***Methods.*** Mother–infant pairs were enrolled at 1 week of life, and then seen at 2- to 3-week intervals through 14 weeks of age. At each visit, nasopharyngeal (NP) swabs were obtained from both, and symptoms were catalogued. Using polymerase chain reaction (PCR) to identify cases, and a severity scoring system to triage these into severe/nonsevere, we calculated disease incidence using person-time at risk as the denominator.

***Results.*** From a population of 1981 infants, we identified 10 with clinical pertussis, for an overall incidence of 2.4 cases (95% confidence interval [CI], 1.2–4.2) per 1000 infant-months and a cumulative incidence of 5.2 cases (95% CI, 2.6–9.0) per 1000 infants. Nine of 10 cases occurred within a 3-month window (May–July 2015), with highest incidence between birth and 6 weeks of age (3.5 cases per 1000 infant-months), concentrated among infants prior to vaccination or among those who had only received 1 dose of Diphtheria Tetanus whole cell Pertussis (DTwP). Maternal human immunodeficiency virus (HIV) modestly increased the risk of infant pertussis (risk ratio, 1.8 [95% CI, .5–6.9]). Only 1 of 10 infant cases qualified as having severe pertussis. The rest presented with the mild and nonspecific symptoms of cough, coryza, and/or tachypnea. Notably, cough durations were long, exceeding 30 days in several cases, with PCRs repeatedly positive over time.

***Conclusions.*** Pertussis is circulating freely among this population of Zambian infants but rarely presents with the classical symptoms of paroxysmal cough, whooping, apnea, and cyanosis. Maternal HIV appears to increase the risk, while lack of effective exposure to DTwP increased the risk.

*Bordetella pertussis*, the agent of whooping cough, infects millions of children around the world and kills hundreds of thousands [1–4]. Pertussis is one of the most contagious of all infectious diseases and the least well prevented of all vaccine-preventable childhood diseases [5]. Severe and fatal cases are concentrated among very young infants between birth and the first few months of life [6], prior to the onset of infant vaccinations and before infants can develop effective endogenous immunity.

Attempts to solve this problem by accelerating the start of the infant vaccine series have been disappointing, due largely to the immaturity of the infant immune system. The most profound deficits are in humoral responses, manifesting as attenuated or absent immune response to vaccines, lower peak antibody concentrations after vaccination, shorter persistence of responses, and lower-affinity antibodies [7, 8]. For example, in a study evaluating the immunogenicity of birth dose pertussis, newborns not only demonstrated poor responses to the birth dose but also had attenuated responses to pertussis vaccines given later as part of the routine infant series [9].

Given the practical limitations of birth dose vaccination, the strategy of administering the adolescent booster vaccine Tdap (tetanus, reduced-dose diphtheria, acellular pertussis) to mothers during pregnancy to augment passive transfer of maternal pertussis antibodies in utero has gained much attention. A recent observational study of maternal Tdap in the United Kingdom showed a high degree of efficacy, and maternal Tdap is now established policy in the United States and the United Kingdom [10]. Unfortunately, the high cost of commercially available Tdap vaccines renders them unaffordable to low- and middle-income countries (LMICs). This has spurred interest in the development of a more affordable pertussis vaccine suitable for a global maternal vaccination strategy.

In preparing the way for the development of such a vaccine, the Bill & Melinda Gates Foundation commissioned a trio of burden-of-disease studies to determine the incidence of severe and nonsevere pertussis in LMICs. The countries selected were Zambia (high human immunodeficiency virus [HIV] prevalence, continued use of infant whole-cell pertussis [wP] vaccine), Pakistan (low HIV prevalence, but also uses wP vaccines), and South Africa (high HIV prevalence, but recently switched to acellular pertussis [aP] vaccine). This article summarizes the findings from the Southern Africa Mother Infant Pertussis Study (SAMIPS) in Zambia and addresses the following questions: (1) What is the incidence of severe and nonsevere pertussis among Zambian infants? (2) What factors explain differences in incidence? and (3) What is the clinical symptomatology associated with these cases?

## METHODS

### Overview of SAMIPS

To provide methodological consistency, the Zambia site harmonized its screening case definition, nasopharyngeal (NP) swabbing procedures, polymerase chain reaction (PCR) testing processes, and pertussis severity scoring systems with the Pakistan and South African sites (see Omer et al and Nunes et al in this supplement).

SAMIPS was a longitudinal birth cohort. The SAMIPS study population consisted of mother–infant pairs from the Chawama compound, a large informal periurban slum located to the southwest of Lusaka's city center. Chawama compound measures roughly 30 km^2^ and is home to a population of approximately 142 000 persons. The Chawama Primary Health Clinic (PHC) is the only government-supported clinic in Chawama compound and the predominant source for all medical care in this community. Housing our study at the Chawama PHC put us at the nexus of nearly all primary care in the compound.

The institutional review boards at Boston Medical Center and Excellence in Research Ethics and Science Converge in Lusaka jointly provided ethical oversight. All mothers provided written informed consent, with consent forms presented in English and the 2 dominant vernacular languages spoken in Lusaka: Bemba and Nyanja. As this was an observational study, it did not need to be registered at ClinicalTrials.gov.

Enrollment was limited to mothers who signed consent and agreed to all procedures, anticipated remaining in the Chawama catchment area for the next 3 months, and granted us access to records documenting their HIV status. To minimize mortality-related attrition in this longitudinal cohort, infants were limited to those who were deemed healthy, were not premature (<37 weeks’ gestation) or underweight (<2800 g), did not result from a complicated pregnancy or delivery, and were within their first 10 days of life. Mother–infant pairs were enrolled at the first postpartum scheduled well-child visit. Baseline data were collected on the mother and infant, including maternal HIV status and CD4 counts if available, maternal age, household composition, infant birth weight, gestational age, and other factors. The study did not measure CD4 counts for HIV-infected mothers, but only used data already available at the Chawama PHC HIV clinic. We did not do confirmatory HIV testing but instead relied on testing previously done at the clinic. Maternal pertussis vaccination status could not be determined given the absence of such records and given that asking mothers to recall their infant vaccination status was unrealistic. While they might have given answers, there would be no way to verify them, and we had little confidence that the subjects could reasonably be expected to know the answer. It was not practical to obtain baseline blood samples from all 2000 mothers to allow retrospective serologic analyses around the subset of infants who might later develop pertussis.

At baseline and thereafter at 2- to 3-week intervals, both members of the mother–infant pair underwent NP sampling. Symptom data were solicited in parallel using a standardized checklist to characterize the subject at each sampling point. The goal of scheduling the visits and obtaining samples irrespective of symptoms was to minimize sampling bias. Ad hoc sick visits also resulted in NP swabs of both members of the pair if the visit was triggered by respiratory complaints in either mother or infant. Clinical data and NP sampling was performed by members of the SAMIPS study team (either a registered nurse or a clinical officer).

Infants received scheduled vaccines at 6, 10, and 14 weeks with the pentavalent vaccine (diphtheria/tetanus/whole-cell pertussis, *Haemophilus influenzae* type b conjugate, and hepatitis B) (Pentavac, Serum Institute of India Limited, Pune, India), the 13-valent pneumococcal vaccine, oral rotavirus vaccine, and oral polio vaccine (OPV). BCG and a first dose of OPV are given at birth, but this occurred prior to enrollment.

### Quality Control of Sample and Data Collection

The fidelity of the sample identification rested on bar codes to uniquely identify subjects and subject data linked to bar codes to uniquely identify samples. The subject and sample ID bar codes were scanned at the time of collection using the Xcallibre digital pen system, which was also used to capture data electronically. For every mother–infant pair, we custom printed 4000 subject ID sticker books, consisting of 50 identical study ID barcode labels per subject (half printed in pink as XXXX-0 for mothers and half in green as series XXXX-1 for infants), and affixed to each case report form as needed. Samples were labeled using a set of 35 000 unique sample ID barcodes, printed in duplicate: 1 copy for the case report with symptoms data and the second for the sample tube. Using this chain of barcodes, we linked subjects to samples with digitally recorded dates, and mated these to each individual's symptom data.

### Laboratory Procedures

NP swabs were obtained using flocked-tipped nylon swabs (Copan Diagnostics, Merrieta, California), sized for adults or infants as indicated. In standardized comparative studies vs comparator NP swabs, flocked-tipped nylon swabs yielded a higher rate of culture positivity, had higher colony-forming unit densities, and yielded higher DNA concentrations on quantitative PCR compared with Dacron or Rayon swabs [11, 12]. Swabs were inserted into both nares until they contacted the posterior nasopharynx and were rotated 180 degrees in both directions. The swabs were then placed in commercially prepared tubes with universal transport media (UTM) and stored on ice until transport. Samples were collected from the study clinic each day and taken to the PCR laboratory at the University Teaching Hospital (UTH) and stored at −80°C until PCR testing.

Our primary analyses were conducted using the diagnostic testing algorithm developed and validated by the respiratory pathogens group at the US Centers for Disease Control and Prevention (CDC) (Supplementary Tables 1*A* and 1*B*). DNA was extracted using the NucliSENS EasyMag system (bioMérieux, Marcy l'Etoile, France) [13]. Pathogen detection was done using a TaqMan genomic assay using the AB7500 Fast Real-Time PCR system (Applied Biosystems, San Francisco, California). Testing starts with a pair of singleplex reactions testing for the targets IS*481* and *ptxS1*. IS*481* is the most common insertion sequence in *B. pertussis*, with multiple copies per genome, making it a very sensitive target for screening [14, 15]. By contrast, *ptxS1*, coding for pertussis toxin, usually exists as a single or occasionally double copy, making *ptxS1* highly specific but less sensitive [16–18]. Because these primers/probes have different annealing temperatures, they were run in parallel on separate 96-well plates. If either IS*481* or *ptxS1* was positive, DNA was reextracted and a multiplex PCR reaction conducted repeating the tests for IS*481* and *ptxS1*, and now including primers/probes specific to *Bordetella parapertussis* (PIS1001) and *Bordetella holmesii* (HIS1001). All primers and probes were purchased from Life Sciences Solutions (a subsidiary of Fisher Biosciences). This paper only provides results for the *B. pertussis* reactions.

Pseudo-outbreaks of pertussis due to accidental contamination of NP swabs at the time of collection, or subsequently in the laboratory, have been reported frequently in recent years [19, 20]. The leading cause of contamination is during sample collection due, ironically, to pertussis vaccines. All wP vaccines used around the world include pertussis DNA, but so too does the leading US multivalent vaccine Pentacel (Sanofi Pasteur). To minimize contamination of our NP swabs during collection, our clinic did not store or administer any vaccines. For infants who required routine vaccinations, these were administered only after all study data and sample collections were complete and then were administered at a location roughly 50 meters away from our clinic, accessed via a separate building entrance. To exclude contamination in the laboratory, all PCR runs included a positive and negative control, the former to confirm that the PCR reaction was successful and generating consistent results across runs, the latter to screen for environmental contaminations within the laboratory. To ensure fidelity of the NP swabs, every patient sample was tested with a primer/probe against the human gene RNAse P. Its product is a constitutive enzyme secreted by all human cells, and therefore tests whether the swab made effective contact with the respiratory mucosa.

### Sample Size Assumptions

For our starting point, we referred to the recently completed Pneumonia Etiology Research in Child Health (PERCH) study. PERCH was a 7-country epidemiologic surveillance study of severe pediatric pneumonia, which included the Lusaka, Zambia, site. PERCH was a hospital-based case-control study, and defined its “cases” as children, aged ≥6 weeks, presenting with a clinical syndrome compatible with severe or very severe pneumonia per World Health Organization (WHO) criteria. Of these, 356 PERCH children were aged 1–6 months, and therefore germane to the SAMIPS estimates. A total of 20 PERCH infants with severe pneumonia tested positive for pertussis by PCR, and 14 of 20 (70%) were aged 1–3 months. Overall, we observed that pertussis accounted for roughly 4% of severe pneumonia in Lusaka infants 1–3 months of age. We extrapolated rates from these data to estimate a population incidence in the relevant age category for SAMIPS using the following assumptions:

- From a cohort of roughly 4000 children admitted to Lusaka's UTH each year, roughly 1750 (44%) had severe or very severe pneumonia.
- If these data were typical, the approximately 4% of children aged 1–3 months with severe or very severe pneumonia proved to have pertussis represent about 1.3% of all children admitted to UTH per year, or approximately 12 per 1000 young infants aged 1–3 months.
- Considered as a rate and assuming equal incidence per month, this yielded an incidence of approximately 6 cases per 1000 infant-months.

Given that this was a hospital-based cross-sectional study, not a true population-based survey, we adopted a more conservative assumption that incidence could be 3 times lower than implied by PERCH. Therefore, taking instead a rate of pertussis of 2 cases per 1000 infants per month as a plausible lower incidence bound, with a margin of error of ±0.2% as the width of the desired confidence interval, and using the sample size formula for a single proportion:

$$n=\frac{[\text{degrees of freedom}*\text{Np(1}-p)]}{[(d^{2}/Z_{1-\alpha /2}^{2}*(N-\text{1)}+p*(\text{1}-p)]},$$

then 1914 subjects would detect this incidence rate for pertussis with 95% confidence. Rounding up, our target sample size was 2000 mother–infant pairs.

### Statistical Analyses

We defined cases as an infant presenting with any of the signs or symptoms on our screening form with a positive PCR result per CDC criteria. Note that this is distinct from the CDC case definition used in routine surveillance in the United States. That definition can be met in 1 of 2 pathways: (1) if an infant has a cough of any duration with microbiological culture confirmation; or (2) if an infant has 2 weeks of cough plus classic symptoms of pertussis (whooping, paroxysms, apnea, posttussive vomiting). In so doing, the CDC's definition increases the specificity of detection while sacrificing sensitivity relative to our screening case definition. Prior to the study start, in discussions with the scientific advisory group, it was decided that PCR was sufficiently persuasive if following the CDC's protocol (which we were). Hence, cultures were not obtained, meaning that there would be no way to satisfy the first pathway. The second pathway is optimized to identify classic pertussis. We note that infants presenting with these symptoms would likely be classified as “severe pertussis” using the Preziosi scale (see below), conflicting with our objective of also measuring nonsevere pertussis.

**Figure 1.**
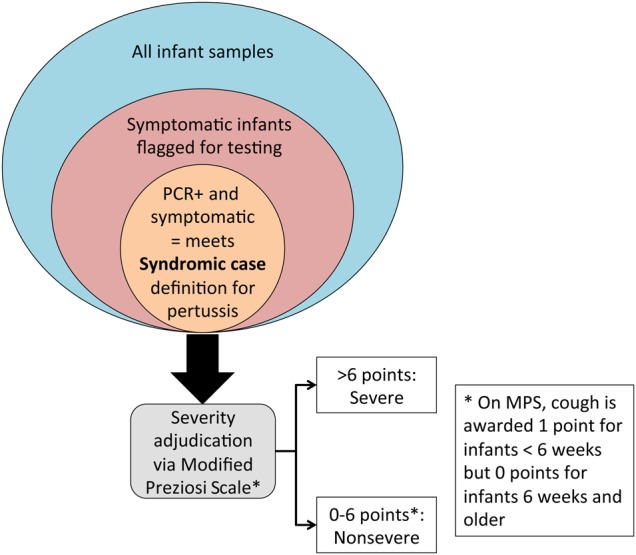
Relationship between the total infant population, the symptomatic population, and those with polymerase chain reaction (PCR)–confirmed pertussis and how these were triaged by severity using the Modified Preziosi Scale (MPS).

For our incidence calculations, we used person-time as the denominator, with infants with PCR-confirmed symptomatic pertussis as the numerators. Positive cases were further classified as severe/nonsevere pertussis using the pertussis severity scoring system developed by Monica Preziosi at the WHO [21–23]. The Preziosi Scale was developed for older children, not infants. Because infants may present with different symptoms than older children, we created a Modified Preziosi Scale (MPS) for use among the infants <6 weeks of age. Symptomatic pertussis was defined as a positive PCR result (per CDC criteria) in the presence of any of our screening symptoms. Nonsevere pertussis was defined as an MPS score of 0–6 points; severe is >6 points. This process is summarized in Figure 1. The MPS is included as Supplementary Table 2.

The enrolled population consisted of those who had at least a baseline visit and NP swab. No imputation was performed for missing data. In summaries, missing data did not contribute to the denominators in means and percentages. Subjects were analyzed to the extent that they made study visits. The following endpoints were calculated: the incidence rate of pertussis (all) and severe and nonsevere pertussis (separately) defined as the number of cases divided by the total person-time per 1000 months. In stratified analyses, we calculated the contribution of maternal and infant characteristics on the above measurements. This included maternal HIV serostatus, CD4 count, and infant ages in months. Additionally, we calculated incidence as a function of the number of pentavalent vaccine doses administered prior to onset of symptoms. For this last analysis only, we defined cases as occurring “postvaccination” if they occurred at least 2 weeks after the latest vaccination, thereby granting sufficient time for the infant to have mounted an immune response. All data manipulations and statistics were performed using SAS software, version 9.4 (SAS Institute, Cary, North Carolina).

## RESULTS

Between March and the end of November 2015, we enrolled 1981 mother–infant pairs (3962 subjects total) out of 3033 pairs screened, for a screening success rate of 65.5%. Reasons for exclusion are provided in Supplementary Table 3. The leading reasons for exclusion were refusal to sign consent (14.3%), low infant birth weight (<2800 g; 7.9%), and nonresidence in the Chawama compound (5.9%). Between March 2015 and February 2016, when the final follow-up visit occurred, we conducted approximately 10 000 study visits and collected approximately 20 000 paired NP swabs.

Tables 1 and 2 summarize the baseline characteristics of the infants (stratified by final pertussis infection status) and their mothers, respectively. Most infants were delivered at the Chawama PHC with a median birth weight of 3000 g (interquartile range [IQR], 2800–3300 g) and enrolled at a median of 7 days postpartum (IQR, 6–8 days). These were similar between infants who later developed pertussis and infants who did not. Among the mothers, 17.5% were HIV infected, of whom 90.6% had received antiretroviral prophylaxis to prevent mother-to-child transmission. No data exist on the proportion of HIV-exposed infants who developed HIV infection during the follow-up period.

**Table 1.** Baseline Demographic Characteristics of the Full Infant Cohort, Stratified by Final Pertussis Infection Status

Parameter	With Pertussis	Without Pertussis	All Subjects
No. of infants	10	1971	1981
Age at enrollment, d, median (IQR)	6.5 (5–8)	7.0 (6–8)	7.0 (6–8)
Place of birth			
UTH	10.0 (1/10)	34.9 (688/1969)	34.8 (689/1979)
Chawama Clinic	90.0 (9/10)	56.8 (1118/1969)	56.9 (1127/1979)
Chilenje Clinic	0.0 (0/10)	1.4 (27/1969)	1.4 (27/1979)
Home delivery	0.0 (0/10)	4.3 (85/1969)	4.3 (85/1979)
Other	0.0 (0/10)	2.6 (51/1969)	2.6 (51/1979)
Male sex	30.0 (3/10)	53.1 (1047/1971)	53.0 (1050/1981)
Birth statistics			
Gestational age			
Estimated gestational age at delivery, wk, median (IQR)	40.0 (38–40)	40.0 (39–40)	40.0 (39–40)
Birth weight, g, median (IQR)	3100 (2800–3300)	3000 (2800–3300)	3000 (2800–3300)
Twin birth	0.0 (0/10)	0.4 (7/1951)	0.4 (7/1961)
Immunizations at birth			
BCG	80.0 (8/10)	46.7 (911/1949)	46.9 (919/1959)
OPV	30.0 (3/10)	33.8 (659/1947)	33.8 (662/1957)
Mother HIV infected	30.0 (3/10)	17.5 (344/1971)	17.5 (347/1981)

Data are presented as percentage (No.) unless otherwise indicated.

Abbreviations: ART, antiretroviral therapy; BCG, bacillus Calmette-Guerin; HIV, human immunodeficiency virus; IQR, interquartile range; OPV, oral polio vaccine; UTH, University Teaching Hospital, Lusaka.

**Table 2.** Baseline Characteristics of Southern Africa Mother Infant Pertussis Study Mothers

Parameter	All Subjects
No. of mothers	1981
Age at enrollment, y, median (IQR)	25.0 (21–29)
Married	90.3 (1787/1978)
Labor and delivery complications	
Obstructed labor	0.1 (2/1902)
Birth asphyxia	1.8 (34/1904)
Sepsis	0.2 (3/1904)
Hemorrhage	0.8 (15/1904)
Other birth complication	1.2 (23/1905)
Maternal immunization	
Received at least 1 dose of TT in current pregnancy	99.5 1971/1981
No. of doses of TT in current pregnancy, median (IQR)	3.0 (2–5)
Maternal HIV status	
Mother HIV infected	17.5 (347/1981)
HIV-infected mother and already receiving ART	90.6 (310/342)
Mother on ART prior to pregnancy	52.0 (157/302)
Trimester initiated ART	
First trimester	13.1 (18/137)
Second trimester	54.0 (74/137)
Third trimester	32.8 (45/137)

Data are presented as percentage (No.) unless otherwise indicated.

Abbreviations: ART, antiretroviral therapy; HIV, human immunodeficiency virus; IQR, interquartile range; SAMIPS, Southern Africa Mother Infant Pertussis Study; TT, tetanus toxoid.

The enrolled infants contributed 4254 person-months of observation time. From the 1981 infants, 775 infants had 1 or more visits with respiratory tract infections, leading to testing of 1165 NP swabs by PCR. From the PCRs on these NP swabs, all *B. pertussis–*positive controls tested positive with cycle threshold (Ct) values typically between 15 and 20 (lower Ct values represent stronger signals; a Ct <40 is considered positive), all negative controls remained negative, and the RNAse P reactions were positive in all cases. A total of 17 positive PCR results identified clinical pertussis in 10 infants (Table 3). The overall pertussis incidence was 2.4 cases per 1000 infant-months (95% confidence interval [CI], 1.2–4.2). Using the maximum severity score for each infected infant, only 1 infant qualified as “severe pertussis” via MPS criteria (incidence rate, 0.2 cases/1000 infant-months), meaning that 90% of infected infants had nonsevere pertussis (incidence rate, 2.1 cases per 1000 infant-months). Parenthetically, we note that the single case of severe pertussis also met the CDC screening case definition for pertussis.

**Table 3.** Incidence of Severe and Nonsevere Infant Pertussis

Pertussis	No. of Infants	Person-time, months	Incidence Rate per 1000 Person-months (95% CI)	Cumulative Incidence per 1000 Infants (95% CI)
All pertussis	10	4254	2.4 (1.2–4.2)	5.1 (2.6–9.0)
Nonsevere pertussis	9	4254	2.1 (1.0–3.9)	4.5 (2.2–8.3)
Severe pertussis	1	4254	0.2 (.1–1.6)	0.5 (.3–2.5)

Abbreviation: CI, confidence interval.

The cases were tightly clustered in time, with 9 of 10 cases occurring between May and July 2015, and a single case in November 2015 (Table 4). During these peak months, incidence tripled to >6 cases per 1000 infant-months. Incidence was highest between birth and the first month of life (incidence rate, 3.5 cases per 1000 infant-months), lower in month 2, and rose again in month 3 (Table 5). When stratifying these based on the number of pentavalent doses administered, and crediting at least 2 weeks from vaccination to allow for seroconversion, incidence rates were quite similar regardless of whether the infants had received 0, 1, or 2 doses of pentavalent vaccine (range, 2.1–2.7 cases per 1000 infant-months; Table 6). The single “severe” case had not yet received any pertussis vaccines. Because the final study visit coincided with the 14-week date when the final pentavalent dose was given, we have no incidence data following pentavalent dose 3. Maternal HIV seropositivity marginally increased the risk of infant pertussis infection, but the difference was not statistically significant (risk ratio, 1.8 [95% CI, .5–6.9]; Table 7).

**Table 4.** Incidence of Pertussis by Calendar Month

Calendar Month	No. of Infants	Person-time, months	Incidence Rate per 1000 Person-months
March 2015	0	69	0
April 2015	0	238	0
May 2015	3	470	6.4
June 2015	3	604	5.0
July 2015	3	573	5.3
August 2015	0	511	0
September 2015	0	441	0
October 2015	0	451	0
November 2015	1	408	2.5
December 2015	0	304	0
January 2016	0	178	0
February 2016	0	7	0

**Table 5.** Incidence of Severe and Nonsevere Pertussis by Infant Age

Age	No. of Infants	Person-time, months	Incidence Rate per 1000 Person-months
All pertussis			
0 mo	4	1128	3.5
1 mo	1	1367	0.7
2 mo	3	1255	2.4
3 mo	2	503	4.0
4 mo	0	1	0.0

**Table 6.** Incidence of Infant Pertussis by Prior Number of Whole-Cell Pertussis Vaccinations

Immunization Status^a^	No. of Infants	Person-time, months	Incidence Rate per 1000 Person-months^b^
All pertussis			
No immunization	4	1882	2.1
1 DTP dose	4	1458	2.7
2 DTP doses	2	914	2.2
Nonsevere pertussis			
No immunization	3	1882	1.6
1 DTP dose	4	1459	2.7
2 DTP doses	2	915	2.2
Severe pertussis			
No immunization	1	1882	0.5
1 DTP dose	0	1463	0
2 DTP doses	0	921	0

Abbreviation: DTP, diphtheria-tetanus-pertussis vaccine.

^a^ The final nasopharyngeal swab was obtained at the same visit at which DTP dose 3 was given. Therefore, we have no incidence data after DTP dose 3.

^b^ In all cases, incidence was calculated allowing for 2 weeks for the infants to respond to the latest pertussis vaccination.

**Table 7.** Incidence of Infant Pertussis as a Function of Maternal Human Immunodeficiency Virus Serostatus

Maternal HIV Status	No. of Women	*Bordetella pertussis* Cases	Person-time, months	Incidence Rate per 1000 Person-months (95% CI)	Cumulative Incidence per 1000 Infants (95% CI)
All women	1981	10	4254	2.4 (1.2–4.2)	5.1 (2.6–9.0)
Positive	347	3	811	3.7 (.9–10.1)	8.7 (2.2–23.5)
Negative	1601	7	3365	2.1 (.9–4.1)	4.4 (1.9–8.75)
Unknown	33	0	78	0.0	0.0
			Risk ratio	1.8 (.5–6.9)	

Of the 3 HIV-infected women with pertussis, we only had reports of 1 of their CD4 counts. It was a value of 545 × 10^3^ cells/mL.

Abbreviations: CI, confidence interval; HIV, human immunodeficiency virus.

Table 8 provides a line-listing summary of the 10 infants with pertussis. Key points of note, 7 of 10 were females, and 3 of the 10 were born to HIV-infected mothers. Eight of the 10 cases were infected, having received zero doses or only 1 dose of pentavalent vaccine, meaning that they probably had little or no vaccine-derived immunity at that point in time. In fact, 4 of the 8 cases were identified at the 6-week visit when the first dose of pentavalent vaccine was to be given.

**Table 8.** Summary Line Listing of the 10 Cases of Infant Pertussis

Subject No.	Sex	Mother's HIV Status	Month of Diagnosis	MPS Score	Age at Diagnosis, wk	No. of DTP Doses Prior to Diagnosis	Days From Last Immunization to Diagnosis
0214-1	Male	Negative	May	3	8	1	17
0424-1	Male	Negative	May	2	2	0	0
0474-1	Female	Positive	May	2	4	0	0
0346-1	Female	Negative	June	1	9	1	20
0714-1	Male	Positive	June	2	4	0	0
0752-1	Female	Negative	June	18	3	0	0
0269-1	Female	Negative	July	2	14	2	25
0579-1	Female	Negative	July	4	12	1	42
0691-1	Female	Negative	July	4	8	1	15
1162-1	Female	Positive	November	1	14	2	31

Abbreviations: DTP, diphtheria-tetanus-pertussis vaccine; HIV, human immunodeficiency virus; MPS, Modified Preziosi Scale.

Most infants presented with mild symptoms, with MPS scores from 1 to 5; the only infant qualifying as severe had an MPS score of 18. This becomes clearer when looking at the case-by-case breakdown of symptoms as summarized in Table 9. Cough and/or coryza were nearly universally present, while tachypnea was observed in 40% of cases. The lack of specificity of cough/coryza/tachypnea is emphasized when observing the ubiquity of these same 3 symptoms among the full SAMIPS infant cohort, >99% of whom did not have pertussis (Supplementary Table 4). The sole infant with severe pertussis (0752-1) presented with an extended duration of cough (≥40 days), several weeks of severe cough, coryza, a history of whoop, observed whooping, observed tachypnea, and signs of bronchial pneumonia on physical examination.

**Table 9.** Reported or Observed Symptoms and Signs Among the Infants With Polymerase Chain Reaction–Confirmed Clinical Pertussis

	Subject ID No.										
Symptom	0214-1	0269-1	0346-1	0424-1	0474-1	0579-1	0691-1	0714-1	0752-1	1162-1	All Subjects
Modified Preziosi Scale score	3	2	1	2	2	4	4	2	18	1	…
Symptom											
Cough (including severe) of any duration	**Yes^b^**	**Yes^b^**	No	**Yes^c^**	**Yes^c^**	**Yes^b^**	**Yes^b^**	**Yes^c^**	**Yes^c^**	**Yes^b^**	90%
Coryza	No	**Yes^c^**	**Yes^c^**	**Yes^c^**	**Yes^c^**	**Yes^c^**	**Yes^c^**	**Yes^c^**	**Yes^c^**	**Yes^c^**	50%
History of whoop	No	No	No	No	No	No	No	No	**Yes^c^**	No	10%
Current whoop	No	No	No	No	No	No	No	No	**Yes^c^**	No	10%
History of posttussive emesis	No	No	No	No	No	No	No	No	No	No	0%
History of cyanosis	No	No	No	No	No	No	No	No	No	No	0%
Witnessed cyanosis	No	No	No	No	No	No	No	No	No	No	0%
History of fits or seizures	No	No	No	No	No	No	No	No	No	No	0%
Witnessed seizures	No	No	No	No	No	No	No	No	No	No	0%
Witnessed tachypnea	**Yes^c^**	No	No	No	No	**Yes^c^**	**Yes^c^**	No	**Yes^c^**	No	40%
Current severe chest indrawing	No	No	No	No	No	No	No	No	No	No	0%
History of lethargy	No	No	No	No	No	No	No	No	No	No	0%
Witnessed lethargy	No	No	No	No	No	No	No	No	No	No	0%
History of difficulty feeding	No	No	No	No	No	No	No	No	No	No	0%
Witnessed poor feeding confirmed by poor suck	No	No	No	No	No	No	No	No	No	No	0%
Measured temperature >38°C	No	No	No	No	No	No	No	No	No	No	0%
History of baby feeling hot or feverish	No	No	No	**Yes^b^**	**Yes^b^**	No	No	No	No	No	20%
History of wheeze	No	No	No	No	No	No	No	No	No	No	0%
Current mechanical sequelae of cough	No	No	No	No	No	No	No	No	No	No	0%
Current conjunctival injection	No	No	No	No	No	No	No	No	No	No	0%
Witnessed paroxysmal cough	No	No	No	No	No	No	No	No	No	No	0%
Witnessed pulmonary signs (bronchitis or pneumonia on examination)	No	No	No	No	No	No	No	No	**Yes^c^**	No	10%
History of difficult or labored breathing	No	No	No	No	No	No	No	No	No	No	0%
History of spells where baby stops breathing	No	No	No	No	No	No	No	No	No	No	0%
Witnessed apnea	No	No	No	No	No	No	No	No	**Yes^c^**	No	10%

^a^ For infants who had >1 positive polymerase chain reaction result, this is the maximum recorded Modified Preziosi Scale (MPS) score. We have bolded all symptoms that were present at the index visit. Not all symptoms are awarded points on the MPS. For infants aged ≥6 weeks, cough is awarded zero points.

^b^ Did not contribute points. In the case of cough, this was because the infant was ≥6 weeks of age.

^c^ Symptoms contributed points to the MPS score calculation.

**Figure 2.**
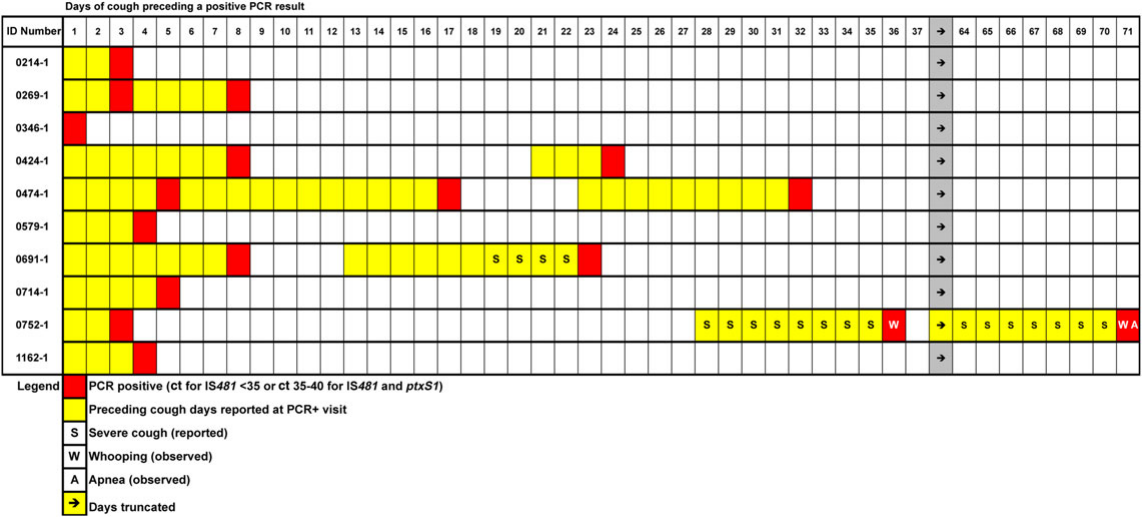
Temporal relationship between days of cough preceding a visit in which the polymerase chain reaction (PCR) result on the nasopharyngeal swab was unequivocally positive per Centers for Disease Control and Prevention criteria (Ct, cycle threshold). Each row corresponds to 1 of the 10 infants with pertussis identified in the Southern Africa Mother Infant Pertussis Study. The results are aligned with the first day being the first day of cough reported for this infant; all subsequent days of cough and PCR results are relative to this first day of cough. These are on an absolute scale, and do not therefore reflect calendar time, meaning that these events did not necessarily overlap in time. As can be seen, extended duration of cough was a common finding among these cases, which led to multiple PCRs being obtained over time, and multiple positive reactions on individuals. In theory, the visits should be spaced 14–21 days apart, but mothers did not necessarily follow this schedule. Our data collection system was not designed to follow cough prospectively until resolution. Rather, if a child presented with cough at a given visit, the duration of cough prior to the visit was recorded. For this reason, cough does not appear to extend to the right of PCR-positive visits. However, this is merely an artifact of the data collection system. Similarly, the days of cough were as reported by the mothers and could not be independently verified. This may account for some apparent gaps. For example, subject 0752-1 appears to have had a 1-day gap between a positive PCR and the resumption of cough. This is most likely due to imperfect recall of the cough duration.

The relationship between cough and PCR positivity is shown in Figure 2. One note of explanation: There are apparent “gaps” where cough symptoms seem to disappear, in all cases immediately following a positive PCR reaction. This is purely an artifact of how our data were collected. The mothers were queried retrospectively about symptoms in their infants at scheduled study visits. Because the clinic staff did not systematically review to see if cough had been reported at the prior visit, they did not record when those previously reported cough symptoms had resolved. In other words, our procedures systematically captured cough duration to the left of a PCR test on this figure, but not to the right of the PCR in this time-series analysis. With that caveat, several observations can be made. First, infants often tested positive for pertussis by PCR repeatedly over time. Second, assuming that cough may well have been continuous between the gaps in our data, cough duration was quite long in many cases. Several infants had cough durations of at least 20–30 days, and 1 for at least 40 days. However, a number of infants only had a few days of cough, and 1 infant presented solely with coryza and no history of cough (subject ID 0346-1) at that or any subsequent visit. Severe cough, reported by the mothers, occurred only twice, including the infant with severe pertussis.

Ten infants died during the study, all due to presumed sepsis and/or acute respiratory infections. As a post hoc analysis, we tested all of the NP samples from these infants and their mothers that had been obtained prior to the terminal event. None of these samples tested positive for pertussis. However, this cannot absolutely exclude pertussis as the cause of death, as all this shows is that at the most recent visit they did not have pertussis. Two infants were hospitalized with nonfatal conditions (abdominal distension and vomiting in one; fever and diarrhea in the second). Neither had a respiratory cause for the hospitalization and did not undergo NP sampling on those occasions.

## DISCUSSION

The SAMIPS data paint a picture of a population in which pertussis is circulating freely but goes unrecognized as such because most cases lack the classic symptoms of whooping, apnea, and paroxysmal cough. To place these results in a more familiar context, if our observed rate is typical for LMICs (and our incidence was comparable to what was reported by our colleagues in South Africa and Pakistan), then these “baseline” rates are comparable to the highest levels reported in the recent California pertussis epidemics [24]. Given Zambia's annual birth cohort of approximately 600 000 infants [25], and the cumulative incidence of 5.2 cases per 1000 SAMIPS infants, this would yield approximately 3000 cases of pertussis a year among infants between birth and 3 months of age. While incidence is not at the scale of other common pediatric diseases, such as respiratory syncytial virus, pertussis is quite prevalent. Contrary to expectations, most cases of infant pertussis were nonsevere, presenting with the nonspecific symptoms of cough and coryza. Moreover, our time-series analysis showed that most cases resolved spontaneously without evolving into severe disease. To note, macrolide antibiotics are not used routinely in Zambia, where the first-line treatment for suspected pneumonia is amoxicillin, an antibiotic with no activity against *B. pertussis*. Thus, this resolution would reflect endogenous immune responses mounted by the infant in response to the infection or, plausibly, to the first dose of wP-containing pentavalent vaccine, in the context of waning maternal antipertussis antibody concentrations.

As anticipated, the majority of cases were clustered within the first 2 months of life. Half of the cases occurred prior to DTP dose 1, and the majority of the remaining cases occurred before dose 2. A single dose of infant DTP vaccine at 6 weeks of age may be insufficient to prevent pertussis infection. Therefore, the cases were concentrated among infants who were either unvaccinated or undervaccinated. Although we did not assess maternal pertussis antibody concentrations at birth, those would result either from natural exposure to circulating *B. pertussis* or the residual effect of an unknown number of wP vaccines that might have been administered 2 decades earlier. In a study in South Africa, prior to a first dose of vaccine at 6 weeks of age, infants had low but detectable concentrations of antibodies against filamentous hemagglutinin and pertussis toxin, which were presumably of maternal origin [26].

All but 1 case occurred within a 3-month window during the 9-month period of surveillance, a reminder that pertussis occurs unpredictably and in epidemic fashion. More robust estimates could be obtained by expanding surveillance over multiyear cycles to account for these fluctuations. Consistent with other studies, we observed a strong female to male predominance among the infants with pertussis [27]. Prior studies have been equivocal regarding the relationship between maternal HIV infection and pertussis [28, 29]. Here, maternal HIV appeared to modestly increase infant incidence, although it did not reach statistical significance. Only 1 mother had a recent CD4 count, precluding inferences about the impact of varying degrees of immunodeficiency. Sample size was clearly a limiting factor, so these results should be interpreted conservatively.

Perhaps the most interesting finding was that 90% of infant pertussis cases were nonsevere, presenting with the nonspecific symptoms of cough and coryza.

A reasonable question then is why these data conflict with prior clinical descriptions of pertussis in infants. One possibility that we should acknowledge is that these could be false-positive results. This could be due to contamination of samples during collection or during the testing itself. However, our use of strict quality control procedures should have minimized this potential. Another possibility is false attribution of *B. pertussis* as the cause of the symptoms if the infant was simultaneously infected with another pathogen, such as a respiratory virus, that was actually responsible for the symptoms. With that said, that issue of false attribution would be far more difficult to parse if this had been a cross-sectional analysis. Here, the longitudinal structure of our data, with infants presenting with prolonged cough and/or coryza with multiple positive PCR results over time, provides an additional level of evidence supporting our hypothesis that these were true cases of nonsevere pertussis. Moreover, our collaborator in Pakistan, following a prospective longitudinal cohort design substantially similar to ours, reached similar conclusions (see Omer et al in this supplement).

So if we accept that the results are probably not due to false positives or false attribution, another plausible explanation for the unusual symptomatology is ascertainment bias. This relates to how we selected samples for testing compared with how this is generally done in accordance with testing guidelines for clinical practice. For example, the CDC's screening guidelines recommend testing for pertussis if a child presents with ≥2 weeks of cough plus whooping and/or apnea, cyanosis, or posttussive vomiting, a constellation of symptoms that meets the definition of “severe pertussis” on the Preziosi Scale. The CDC criteria are intended to maximize the signal-to-noise ratio (ie, to maximize specificity at the expense of sensitivity) when used for routine disease surveillance.

While this is intended to reduce false-positive rates, it has the unintended consequence of introducing a logical circularity: by only testing individuals who are clinically suspected to have severe pertussis, all cases so found will, by definition, have severe pertussis. That approach to screening answers the epidemiologic question, “What proportion of infants who look like they might have severe pertussis actually do have severe pertussis?” But that is a fundamentally different question from “What proportion of infants with pertussis have or go on to develop severe pertussis?” And that is because the full spectrum of disease presentations has not previously been assessed systematically and longitudinally. SAMIPS was designed to identify cases across the full spectrum of severity and to observe the evolution of symptoms over time. We tested at the first sign of respiratory symptoms, hence, 100% of our cases had cough durations <2 weeks before the first positive PCR because we saw our clients at 2-week intervals. But the upside to this approach is that it also allowed us to observe the proportion that later developed severe disease (and would presumably have been the kinds of individuals detected per CDC guidelines). In addition, it allowed us to see whose symptoms resolved spontaneously without developing into severe disease.

In conclusion, pertussis was prevalent in this population of Zambian infants. However, such cases will systematically evade clinical diagnosis as they lack the classic symptoms commonly equated with pertussis in infants and children. The preponderance of mild disease identified here seemingly conflicts with prior reports that pertussis infants is usually severe, but this is almost certainly an artifact of ascertainment bias in how samples are selected for testing and the absence of prior longitudinal burden of disease surveillance studies. Further research is needed to clarify the impact of maternal HIV status on infant pertussis. The clustering of pertussis cases within the first few months before effective immunity from infant vaccination can reasonably be achieved supports the rationale for a maternal Tdap vaccination strategy.

## Supplementary Data

Supplementary materials are available at http://cid.oxfordjournals.org. Consisting of data provided by the author to benefit the reader, the posted materials are not copyedited and are the sole responsibility of the author, so questions or comments should be addressed to the author.

Supplementary Data
