# Supplementary material for: Incidence of Severe and Nonsevere Pertussis Among HIV-Exposed and -Unexposed Zambian Infants Through 14 Weeks of Age: Results From the Southern Africa Mother Infant Pertussis Study (SAMIPS), a Longitudinal Birth Cohort Study
Source: Clin Infect Dis. 2016 Nov 2;63(Suppl 4):S154–64. doi: 10.1093/cid/ciw526 (PMC5106616; doi:10.1093/cid/ciw526)
Supplement: Supplementary Data [file supp_63_suppl-4_S154__index.html]

Supplementary Data 

# Incidence of Severe and Nonsevere Pertussis Among HIV-Exposed and -Unexposed Zambian Infants Through 14 Weeks of Age: Results From the Southern Africa Mother Infant Pertussis Study (SAMIPS), a Longitudinal Birth Cohort Study

## Supplementary Data

Supplementary Data

- Supplementary Data - Pdf file
