# Supplementary material for: Incidence of Severe and Nonsevere Pertussis Among HIV-Exposed and -Unexposed Zambian Infants Through 14 Weeks of Age: Results From the Southern Africa Mother Infant Pertussis Study (SAMIPS), a Longitudinal Birth Cohort Study
Source: Clin Infect Dis. 2016 Nov 2;63(Suppl 4):S154–64. doi: 10.1093/cid/ciw526 (PMC5106616; doi:10.1093/cid/ciw526)
Supplement: Supplementary Data [file supp_ciw526_ciw526supp.pdf]

Supplementary Table 1a. Diagnostic algorithm for interpreting PCR results

Primer reactions				Interpretation
IS481	PTxS1	PIS1001*	HIS1001*	
Ct<35	(+) or (-)	NA	NA	*B. pertussis*
35<=CT<40	(+)	NA	NA	*B. pertussis*
35<=Ct<40	(-)	NA	NA	Indeterminate
(+ at any CT value <40)	(-)	(-)	(+)	*B. holmesii*
(-)	(+)	(+)	(-)	*B. parapertussis*
(-)	(-)	(-)	(-)	Non-Bordetella respiratory disease

Abbreviations: CT – Cycle Threshold

Supplementary Table 1b. Primer/probe used sequences for detection of *B. pertussis*: Insertion sequence 481 (IS481) and Pertussis Toxin (ptxS1)

**IS481**
Forward 5'-CAAGGCCGAACGCTTCAT-3'
Reverse 5'-GAGTTCTGGTAGGTGTGAGCGTAA-3'
Probe 5'-NED-CAGTCGGCCTTGCGTGAGTGGG-MGB-3'
**ptxS1**
Forward 5'-CGCCAGCTCGTACTTC-3'
Reverse 5'-GATACGGCCGGCATT-3'
Probe 5'-VIC-AATACGTCGACACTTATGGCGA-MGB-3'

Supplementary Table 2. Components of the Modified Preziosi Scale

Modified Preziosi scale components	Point value
**For infants ≥ 6 weeks**	
Cough plus ≥1 of the following – note, no restriction by duration	
Observed apnea	6
Reported apnea	1
Death**	6
Typical paroxysm with whoop	4
Typical paroxysm, no whoop	3
Pulmonary signs (bronchitis or bronchopneumonia on exam)*	3
Mechanical complication of cough: sub-conjunctival hemorrhages or umbilical or inguinal hernia	3
Seizure	3
Tachypnea RR>50	3
Chest retractions	3
Cyanosis	3
Conjunctival injection	2
Post tussive vomiting	2
Coryza	1
Fever ≥ 38C	1
**For infants < 6 weeks**	
Any of the following symptoms	
Observed apnea	6
Reported apnea	1
Death**	6
Typical paroxysm with whoop	4
Typical paroxysm, no whoop	3
Pulmonary signs (bronchitis or bronchopneumonia on exam)	3
Mechanical complication of cough: sub-conjunctival hemorrhages or umbilical or inguinal hernia	3
Seizure	3
Cyanosis	3
Tachypnea RR>60	3
Chest retractions	3
Lethargy, or movement only when stimulated	3
Poor feed as confirmed by poor suck	3
Conjunctival injection	2

Post tussive vomiting	2
Coryza	1
Cough	1
Fever ≥ 38C	1
Upper respiratory symptoms, NOS	1

Supplemental Table 3. Enrollment summary

**Reason for Exclusion***	**Percent excluded**
Mother does not consent to study procedures and visit schedule	14.3% (428/3,001)
Low birth weight (<2800 grams)	7.9% (236/2,979)
Not a resident of Chawama compound	5.9% (176/3,001)
Mother anticipates moving out of compound in next 6 months	4.1% (123/2,979)
Mother not 18-39 years old	3.7% (110/3,002)
Premature infant	2.9% (87/2,978)
Premature and low birth weight	2.7% (82/3,033)
Mother not presenting within 14 days post-partum	1.6% (49/3,001)
Infant has medical conditions that interfere with ability to complete study	0.6% (18/2,998)
Mothers HIV status is not documented or unwilling to undergo HIV testing	0.3% (8/2,997)
Concern about mother by local investigator	0.2% (6/2,979)
Known serious birth complications	0.2% (5/2,979)
Concern about infant by local investigator	0.1% (4/2,973)
Mothers use during pregnancy of immunosuppressive agents	0.1% (2/2,979)
Uncontrolled post-partum depression	0.0% (0/2,979)

**Summary**	
Number of mothers screened	3,033
Number of mothers eligible for enrollment	1,987
Number of mothers enrolled	1,981
Number of mothers excluded	1,046
Proportion screened and enrolled	65.3%
Proportion screened and excluded	34.5%

***** Categories are not mutually exclusive.

Supplemental Table 4. Proportion of all infants presenting with symptoms at any time*

Symptom	All infants (N=1,981)
Cough (including severe) of any duration	32.6% (645/1,981)
Coryza	36.4% (722/1,981)
History of Whoop	0.3% (5/1,981)
Current Whoop	0.1% (1/1,981)
History of Post-tussive emesis	1.3% (26/1,981)
History of Cyanosis	0.1% (2/1,981)
Current Cyanosis	0.0% (0/1,981)
Current Seizures	0.2% (3/1,981)
History of fits or seizures	0.3% (5/1,981)
Tachypnea	67.9% (1,346/1,981)
Current severe chest in-drawing	1.0% (19/1,981)
History of lethargy	0.2% (3/1,981)
Currently lethargic	0.1% (2/1,981)
History of difficulty feeding	1.5% (29/1,981)
Current poor feeding confirmed by poor suck	0.8% (16/1,981)
Temperature > 38	4.1% (82/1,981)
History of baby feeling hot or feverish	8.8% (175/1,981)
History of wheeze	0.9% (17/1,981)
Current mechanical sequellae of cough	0.0% (0/1,981)
Current conjunctival Injection	0.1% (2/1,981)
Current paroxysmal cough	0.0% (0/1,981)
Current pulmonary signs (bronchitis or pneumonia on exam)	0.4% (7/1,981)
History of difficult or labored breathing	0.7% (13/1,981)
History of spells where baby stops breathing	0.2% (4/1,981)
Current apnea	0.1% (1/1,981)

***** These data include all infants enrolled in SAMIPS, regardless of final pertussis infection status. Some infants may have contributed more than one symptom and at more than one time over the period of observation. These data are not mutually exclusive.
